# A novel frameshift variant in *ALS2* associated with segmental axonopathy in Merino sheep

**DOI:** 10.1186/s12711-025-01005-w

**Published:** 2025-10-23

**Authors:** Katie L. M. Eager, Robert D. Jolly, Leah Manning, Cali E. Willet, Russell G. Snell, Klaus Lehnert, Natasha E. Mckean, Nick W. Sneddon, Brendon A. O’Rourke, Keren E. Dittmer, Imke Tammen, Matt Littlejohn

**Affiliations:** 1 Sydney School of Veterinary Science, The University of Sydney, 425 Werombi Road, Camden, 2570 Australia; 2 Elizabeth Macarthur Agricultural Institute, NSW Department of Primary Industries and Regional Development, 240 Woodbridge Road, Menangle, 2568 Australia; 3 School of Veterinary Science, Massey University, University Avenue, Palmerston North, 4410 New Zealand; 4 Sydney Informatics Hub, The University of Sydney, 1 King Street, Newtown, 2042 Australia; 5 School of Biological Sciences, University of Auckland, 3A Symonds Street, Auckland, 1010 New Zealand; 6 School of Agriculture and Environment, Massey University, University Avenue , Palmerston North, 4410 New Zealand

## Abstract

**Background:**

Segmental axonopathy is a recessively inherited neurodegenerative disorder that has affected Merino sheep since the early 1930s. Despite its long-standing recognition, the genetic basis of the condition remained unknown. This study aimed to identify the genetic cause of segmental axonopathy and confirm its pathological features to improve diagnostic accuracy and inform breeding strategies.

**Results:**

Whole genome sequencing and genotyping of affected and unaffected Merino sheep identified a novel homozygous frameshift variant in the *ALS2* gene that segregated with disease. RNA sequencing of cerebellar peduncle tissue confirmed the nonsense consequence on the *ALS2* transcript. Histological analysis highlighted the hallmarks of the disease as large, foamy eosinophilic axonal swellings predominantly in the trigeminal ganglia, with additional degenerative changes in both the brain and spinal cord. These findings support the value of targeted sampling of sensory roots of the trigeminal nerve, spinal cord tracts, and dorsal nerve rootlets to enhance diagnostic accuracy. The same *ALS2* variant was found across multiple unrelated flocks in both Australia and New Zealand, indicating a broader presence within the fine-wool Merino sheep population.

**Conclusions:**

This study identifies a novel *ALS2* frameshift variant associated with segmental axonopathy in Merino sheep and provides both genetic and histological evidence supporting its role in disease pathology. The development of a DNA diagnostic test will enable more informed breeding decisions, reduce the prevalence of this condition, and improve animal welfare and productivity in the Merino industry. Moreover, the findings offer a potential large-animal model for exploring early-onset forms of human motor neuron diseases, including amyotrophic lateral sclerosis, in which *ALS2* variants are implicated.

**Supplementary Information:**

The online version contains supplementary material available at 10.1186/s12711-025-01005-w.

## Background

Neurological diseases in sheep can have several aetiologies, including plant intoxications, copper or vitamin A deficiency, bacterial or viral infections and transmissible spongiform encephalopathy (TSE). Many suspected or confirmed inherited neurological diseases in sheep are listed in Online Mendelian Inheritance in Animals (OMIA) (https://omia.org/results/?gb_species_id=9940&categories=26&search_type=advanced) [1]. Extensive farming practices, where individual animals are often not monitored daily, and particularly if there is delayed onset of clinical signs, hinder the early detection of neurological issues within a flock. This challenge is further compounded by the difficulty in diagnosing inherited neurological diseases with slow progression without regular, individual animal assessments. Consequently, producers may remain unaware of such diseases in their flock until the condition has significantly progressed.

A progressive, degenerative, neurological disease occurring in Merino sheep, previously reported as segmental axonopathy, Murrurundi disease, and Mudgee ataxia, has been described for more than 80 years (OMIA:001492–9940). Affected sheep have progressive hindlimb weakness, knuckling of fetlocks, ataxia and paresis [2–4], and either die of misadventure or are euthanised [2–5]. Segmental axonopathy is a recessive, late-onset disease that manifests first clinical signs between 1 to 5 years of age and progresses slowly to severe disease [5].

The well-described pathological characteristics of segmental axonopathy allow for clear differentiation from other non-genetic and genetic neurological diseases. While there are no gross lesions of the nervous system, there are detailed histopathological changes [2–7]. Histologically, segmental axonopathy is characterized by large pale eosinophilic foamy swellings in the white matter of the central and peripheral nervous systems [2–4]. Although they may be found throughout the central nervous system, they have a particular predilection for the dorsal rootlets of some sensory nerves, the dorsal funiculus where these enter the cord, in the fasiculus gracilis, the trigeminal nerve root, the dorsal spinocerebellar tract and the superficial transverse tracts of the pons where they extend into the middle cerebellar peduncles. Wallerian degeneration and intra-myelinic oedema also occur in white matter and nuclei, along with peripheral nerve Wallerian degeneration and Schwann nuclei proliferation. Electron microscopy showed that the axonal swellings consisted mainly of membrane bound vesicles derived from in-pouching of myelin [4, 5].

The combined clinical presentation and detailed histopathology has shown that segmental axonopathy affects multiple unrelated flocks across both Australia and New Zealand. This disease is therefore anticipated to significantly contribute to welfare and production consequences within the industry. This paper reports the identification of the likely causal variant for segmental axonopathy and offers a comprehensive discussion of the results, drawing comparisons with similar diseases in humans and other animals.

## Methods

### Animals, sample collection and DNA extraction

Reports and samples from affected animals in 19 Australian flocks and 3 New Zealand flocks were available through Elizabeth Macarthur Agricultural Institute (EMAI) and Massey University as part of diagnostic disease investigations. At the time of tissue collection, the aetiology of the condition was still unknown. Due to animal ethics considerations and uncertainty regarding the carrier status of the flock/s, it was determined that euthanising healthy animals solely for research purposes could not be justified.

Blood and tissue samples were available from 46 clinically suspected affected, 42 related animals and 123 unrelated Merino animals for DNA extraction. Frozen fresh tissue and blood samples were extracted using the DNeasy Blood and Tissue kit (QIAGEN, CA, USA). Formalin fixed paraffin embedded blocks were extracted using QIAamp DNA FFPE Advanced Kit (QIAGEN, CA, USA), following the manufacturer’s protocols.

### Whole genome sequencing and informatics

Whole genome sequencing was performed on two affected Australian animals and an additional animal with an uncertain diagnosis, as well as on three affected and two control sheep from New Zealand. The animal with an uncertain diagnosis was a historical case in which segmental axonopathy was considered a differential diagnosis; however, histopathological review conducted during this study did not reveal the classic lesions associated with segmental axonopathy. For the Australian animals, sequencing libraries were prepared by an external service provider and sequenced on either an MGISEQ-2000RS or an Illumina NovaSeq 6000 instrument (Genewiz, China). Sequencing libraries for New Zealand samples were generated using the Illumina DNA Prep Tagmentation kit and sequenced on an Illumina Novaseq 6000 instrument using a 2 × 150 bp read configuration (Genemark, Hamilton, NZ).

Prior to mapping, sequence read data were processed using Trimmomatic v0.39 [8]. Resultant read data were then mapped to the ARS-UI_Ramb_v2.0 genome assembly using bwa-mem2 v2.21 [9]. All eight samples yielded a mapping rate of > 99%, with read depths ranging between 18.6 and 47.9X depth (mean 28.3X). GATK Haplotypecaller v4.2.4.1 [10] was then used to call variants across these samples, discovering 32,982,076 variants. Application of generic hard filters based on GATK guidelines [11] yielded 28,769,668 variants, with these data then used for segregation filtering and genetic analysis.

Following identification of variants segregating with disease status, functional prediction of these candidates was performed using SNPEff v5.1 [12] in conjunction with RefSeq annotation release 104 of the ARS-UI_Ramb_v2.0 assembly (GCF_016772045.1). Interpretation of these data in conjunction with International Sheep and Goat Consortium (ISGC) 1000 Sheep Genomes information [13] required liftover of ARS-UI_Ramb_v2.0 positions to the Oar_v3.1 assembly. This step was performed using the NCBI-hosted web-based remapping tool remap.

### SNP genotyping, GWAS analysis and homozygosity mapping

Samples from 10 affected Australian Merino sheep and 98 Australian Merino control sheep that were submitted for other disease investigations were genotyped using the Illumina Ovine SNP 600 K BeadChip array (Neogen, USA). Genome-wide association studies (GWAS) and homozygosity mapping were performed using a logistic regression model in PLINK v1.9 [14]. Manhattan plots were generated using qqman (v0.1.9) [15] and runs of homozygosity plotted by detectRUNS (v0.9.6) (https://github.com/bioinformatics-ptp/detectRUNS/tree/master/detectRUNS) in Rstudio, R version 4.2.3v0.1.9.

### RNA extraction, sequencing and informatics

RNA-seq was used to assess the structure of the transcripts in one affected animal. RNA extraction was performed on a small section of RNAlater (Thermo Fisher Scientific, USA) stabilised, left cerebellar peduncle using a 30 mg slice excised on dry ice and lysed with a 2 mm ball bearing, 600 µL of RLT buffer (QIAGEN, Germany) and a TissueLyser II instrument (QIAGEN, Germany) at 25 Hz, for 2 min intervals × 4, swapping position of the sample at each interval. The ball bearing was removed, and the lysed tissue processed through the RNeasy Mini Kit (QIAGEN, Germany), following the manufacturers protocol. The sample was eluted in 50 µL of RNase free water, quantified, and quality evaluated with the Agilent RNA 6000 nano kit on the bioanalyzer (Integrated Sciences, Australia).

A sequencing library was prepared from total RNA by Macrogen (Seoul, Republic of Korea) using the TruSeq Stranded mRNA Library Prep kit (Illumina < USA) and sequenced on a NovaSeq 6000 System to produce 163 million paired-end 150 bp reads. Reads were aligned to the Ovis aries ARS-UI_Ramb_v2.0 genome assembly (GCF_016772045.1; annotation release 104) with STAR 2.7.9a in 2-pass mode [16]. Read alignments were visually inspected with the Integrated Genome Viewer [17] to confirm transcript inclusion of the *ALS2* variant.

### qPCR validation of variant

A custom TaqMan assay (Applied Biosystems, USA) was developed to genotype the *ALS2* variant. The qPCR was performed on a ViiA 7, StepOne or 7500 system (Applied Biosystems, USA) in a final reaction volume of 12.5 µL. The reaction mix consisted of 1 × TaqMan Genotyping Master Mix (Applied Biosystems, USA), 900 nmol/L of allele specific primers 5′-TGCAACCAGAGTCTCCACAAG-3′ and 5′-GCTGCACTGGTTCTCTTCCT-3′, 250 nmol/L of allele specific probes 5′-VIC-ACTCCTCTGCACCCGC-NFQ-3′ (wildtype) and 5′-FAM-CACTCCTGCACCCGC-NFQ-3′ (mutant) and approximately 5–50 ng of genomic DNA. Cycling parameters were pre-read hold at 60 °C for 30 s, initial denaturation at 95 °C for 10 min, followed by 45 cycles of denaturation at 95 °C for 15 s, annealing and extension at 60 °C for 60 s and a final post-read stage at 60 °C for 30 s.

### Protein alignment and in-silico predictions

Protein sequences were sourced from UniProt or NCBI protein and aligned using Multiple Sequence Comparison by Log-Expectation (MUSCLE) [18] in Geneious Prime v2023.2.1 (Dotmatics, UK). Sequences were named using the convention accession_common name. MutPred-LOF predictions [19] were generated using the web server at http://mutpred2.mutdb.org/mutpredlof/index.html by submitting the wild-type and the expected altered transcript sequences based on the identified variant.

### Histopathology

Cases of possible segmental axonopathy from Elizabeth Macarthur Agricultural Institute (EMAI), Australia were found by review of the database of diagnostic submissions from 2011 to 2020 in which axonopathy was diagnosed, as well as cases nominated from diagnostic pathologists in which segmental axonopathy was considered a differential diagnosis. Blind review of signalment, history and histopathology were performed by a single veterinary pathologist to classify cases as likely or unlikely affected with segmental axonopathy, or equivocal if insufficient tissue was examined and segmental axonopathy was unable to be excluded. Tissue samples had previously been processed and embedded in paraffin wax and 4 µm sections cut and stained with haematoxylin and eosin (H&E). The results of the review were then compared with the results from the TaqMan genotyping.

Similarly, slides from cases diagnosed as segmental axonopathy from Massey University, New Zealand (NZ) were reviewed by a veterinary pathologist and the results then compared with genetic testing.

## Results

### Genome and transcriptome sequencing implicate an *ALS2* null allele underlying segmental axonopathy

Whole genome sequencing was performed on five affected animals, two wildtype controls, and one animal of uncertain disease status, followed by read mapping and variant calling based on the ARS-UI_Ramb_v2.0 genome assembly. A total of 28,769,668 variants were eligible for analysis after the application of basic quality filters.

As no strong candidate gene was proposed, all genome variants were considered as potential candidates. Filters were applied assuming a recessive mode of inheritance, excluding all variants that were not homozygous for the alternate allele in all five affected animals, and were not homozygous reference or heterozygous in the two control animals. No zygosity filters were applied to the uncertain status animal. These filters yielded a total of 51,236 genome-wide variants segregating with disease status. Greater than 20% (N = 10,899) of these candidates mapped to chromosome 2. Effect prediction highlighted 79 variants with ‘MODERATE’ or ‘HIGH’ impacts (broadly equating to missense and nonsense classes respectively), with > 20% (N = 18) of these located on chromosome 2. Additional File 1, Table S1 shows the positions and properties of these variants.

**Fig. 1:**
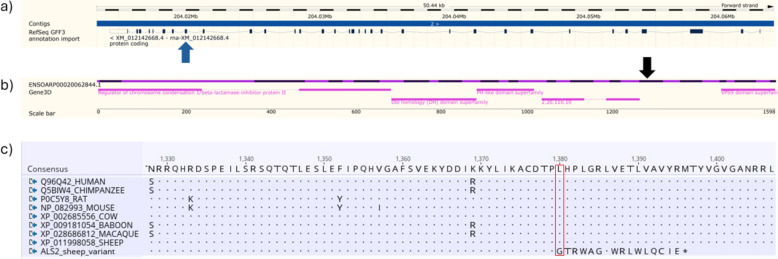
Predicted impact of the *ALS2* frameshift variant in sheep. **a** Schematic of the ovine *ALS2* gene on chromosome 2, showing intron–exon structure (image adapted from the Ensembl genome browser; Ensembl release 114, *Ovis aries* reference genome ARS-UI_Ramb_v2.0). The location of the 2 bp deletion in exon 26 (XM_012142668.4:c.4138-4139del) is indicated by a blue arrow. **b** Diagram of the major functional domains of the predicted ALS2 protein (adapted from the Ensembl genome browser; Ensembl release 114), illustrating how the introduction of a premature stop codon (XP_011998058.1:p.(Leu1380Glyfs*17), position indicated by black arrow, is expected to truncate the downstream VPS9 domain on the far right. **c** Protein alignment of partial ALS2 orthologues from selected species, generated using MUSCLE, highlighting a conserved protein region. The red box marks the predicted frameshift, which introduces a premature stop codon (XP_011998058.1:p.(Leu1380Glyfs*17))

To further refine the list of candidate functional variants, these 79 positions were intersected with population data from the International Sheep Genomics Consortium (ISGC). Here, variant frequency data were obtained from 935 genomes representing 70 different breed categories included as part of Run2 of the 1000 sheep genomes project [13]. As this dataset was aligned to the earlier Oar_v3.1 assembly, the coordinates of the 79 putative deleterious variants, originally mapped to the ARS-UI_Ramb_v2.0 assembly in this study, were converted to Oar_v3.1 for comparison. Seventy-four of the 79 candidate variants were successfully repositioned. Variants with a high allele frequency in the wider population (> 5%) were considered unlikely to be causal for severe disease. Two candidate variants remained following this population filtering. One of the two remaining candidate variants presented 10 alternate allele homozygotes in the 935 genome dataset and was carried by diverse and multiple breeds [See Additional File 1, Table S1] and was therefore also considered unlikely to be causing the phenotype observed in Merino sheep. The final candidate was a 2 bp deletion in the *ALS2* gene on chromosome 2 (NC_056055.1:g.204020069-204020070del, XM_012142668.4:c.4138-4139del, OMIA variant:1828) and was only present in heterozygous form in two sheep in the 935 genome dataset. Notably, both heterozygous animals were also Merino sheep. This frameshift is predicted to cause a leucine to glycine substitution at the mutated codon in exon 26 (Fig. 1a) and introduction of 16 new amino acids prior to the insertion of a premature stop codon (XP_011998058.1:p.(Leu1380Glyfs*17)). If translated, this new sequence would replace the 287 C-terminal residues of the wildtype *ALS2* protein and truncate the VPS9 domain superfamily (Fig. 1b). *ALS2* codes for the protein alsin, which is highly expressed in the nervous system, and variants of this gene in humans are associated with motor neuron diseases [6, 7], thus making this a strong functional candidate variant.

The *ALS2* variant was predicted to cause loss of function of the alsin protein with moderate probability (score = 0.366) by MutPred-LOF [19]. This in-silico analysis also indicates that the frameshift variant may impact several important protein functions, including a catalytic site (p = 0.004076), a protein–protein interaction hotspot (p = 0.0088539), and the ability of alsin to bind iron (p = 0.0010397). Additionally, the predicted protein sequence of the frameshift variant was compared to select protein orthologues, indicating that the variant is located in a conserved area of the gene [Fig. 1, panel C]. The evolutionary conservation of the region in which the variant lies further supports its pathogenicity.

In addition to the frameshift variant in *ALS2*, a missense variant in exon 3 of the same gene was also identified (XM_012142668.4:c.215A > G; p.(Asn72Ser), see Additional File 1, Table S1). This second variant was present at high frequency in the ISGC population dataset, in multiple breeds and in homozygous state in presumed healthy animals. The highest minor allele frequency reported in Ensembl is 0.50 (rs159560565). Sorting tolerance from intolerance (SIFT) analysis [20] returned a score of 1, indicating a predicted tolerated effect on protein function. In ClinVar, the same position is reported as NM_020919.4(*ALS2*):c.215G > A; p.Ser72Asn (National Center for Biotechnology Information. ClinVar; [VCV001441610.7], https://www.ncbi.nlm.nih.gov/clinvar/variation/VCV001441610.7 (accessed Aug. 7, 2025) and is classified as a variant of uncertain significance in infantile-onset ascending hereditary spastic paralysis. However, in sheep this represents the opposite reference allele and amino acid state.

### SNP chip genotyping data supports causality of the *ALS2* variant

**Fig. 2:**
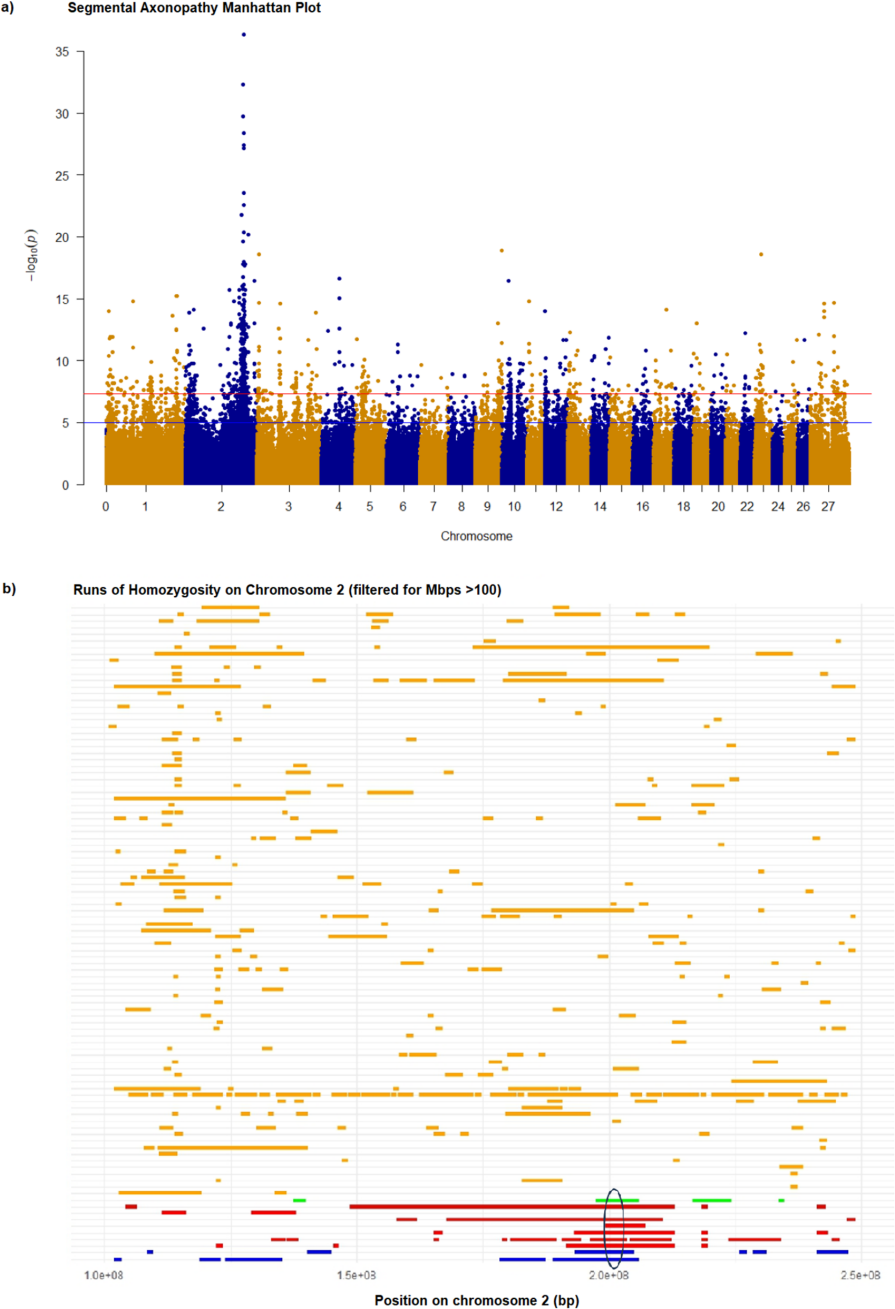
GWAS and ROH analysis implicate *ALS2* as a positional candidate gene for segmental axonopathy in Merino sheep. **a** Manhattan plot of genome-wide association results generated using PLINK, displaying –log₁₀(p) values from a single-marker analysis for segmental axonopathy. Each point represents a SNP, plotted by chromosomal position (x-axis) and strength of association (y-axis). The blue line denotes the suggestive significance threshold (–log₁₀(p) = 5), and the red line marks the genome-wide significance threshold (–log₁₀(p) = 8). **b** Homozygosity mapping on chromosome 2 showing runs of homozygosity > 100 Mbp in 10 affected and 98 unaffected control Merino sheep. A shared homozygous region containing the *ALS2* gene (circled in black) is observed in affected individuals. Orange bars indicate control animals, while green, red, and blue bars represent affected animals from three separate flocks

To independently confirm *ALS2* as a positional candidate gene for segmental axonopathy, we next performed a genome wide association study (GWAS). This analysis used data from 10 affected animals and 98 control Merino sheep collected as part of other studies in our laboratory. A single prominent peak at the distal end of chromosome 2 was found using the logistic regression model implemented in Plink software (v1.9) (Fig. 2a). This was the same region identified from sequence-based analysis, with the top 10 associated variants flanking the *ALS2* frameshift [See Additional file 2, Table S2]. Further, homozygosity mapping showed only one shared region of homozygosity among 9 of the 10 affected animals, also located at the distal end of chromosome 2 between 199,143,511 and 203,625,034 on reference Oar_v3.1 (GCF_000298735.1) (Fig. 2b). Both results support *ALS2* as a positional candidate gene and the identified frameshift as likely responsible for the disease.

### Validation of the *ALS2* variant using RNA sequencing

Given that a disproportionate number of nonsense predictions are due to annotation errors, the next step was to validate the sheep *ALS2* transcript structure and likely consequence of the *ALS2* 2 bp deletion. To this end, RNA sequencing of a cerebellar peduncle sample from an affected animal was conducted and mapped to the ARS-UI_Ramb_v2.0 genome. The alignment of RNA-seq reads of this sample shows that the 2 bp deletion (XM_012142668.4:c.4138-4139del) is present in spliced reads of the *ALS2* transcript [See Additional file 3, Figure S1], confirming the prediction based on the annotated reference genome.

### Validation that the *ALS2* variant segregates with the SA phenotype

**Fig. 3:**
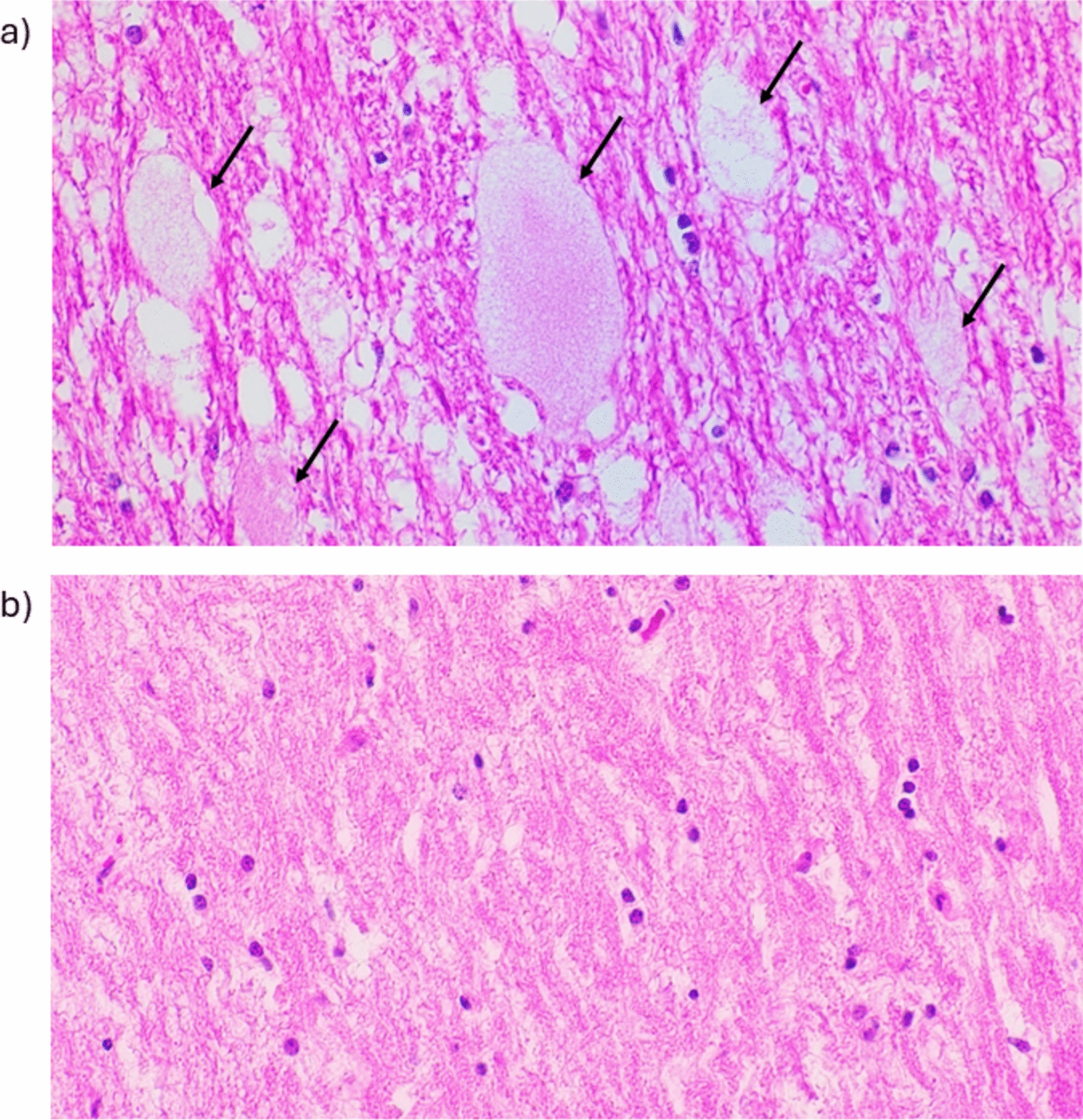
Representative histopathology images from the sensory root of the trigeminal nerve in affected and unaffected Merino sheep. Haematoxylin and eosin (H&E), 40 × magnification. **a** Affected Merino sheep homozygous for the 2 bp deletion in *ALS2* (XM_012142668.4:c.4138-4139del), showing large, foamy, eosinophilic swellings (black arrows) characteristic of segmental axonopathy. **b** Unaffected control Merino sheep homozygous for the wild-type *ALS2* allele

A retrospective review of all cases submitted to the Elizabeth Macarthur Agricultural Institute (EMAI) and Massey University, New Zealand was also performed, where segmental axonopathy was considered as a differential diagnosis. A total of 31 Australian cases and 10 New Zealand cases were reviewed. Cases were classified as likely affected with segmental axonopathy based on the presence of the large, foamy, eosinophilic axonal swellings in the brain and/or spinal cord (Fig. 3a).

To confirm the genetic basis of the disease, a SNP genotyping assay was developed and applied to all reviewed animals with available DNA samples. This assay demonstrated that all 18 histologically confirmed affected animals were homozygous for the *ALS2* variant. In contrast, cases previously suspected of segmental axonopathy based solely on white matter vacuolation and Wallerian degeneration, without the characteristic eosinophilic axonal swellings, were wildtype. Screening of 33 rams from an Australian property with affected animals identified six heterozygous carriers of the *ALS2* variant. However, none of these heterozygous rams sired affected offspring within the flock (in-house parentage data not shown). Similarly, in a New Zealand flock with a history of affected animals, two additional clinically unaffected animals were identified as heterozygous carriers of the variant.

Further examination of genotype calls from the ISGC Run2 sequence dataset [13] revealed that two of the 128 Australian Merinos included in the dataset were heterozygous for the *ALS2* variant, with the variant absent in other breeds. To extend these findings, the SNP genotyping assay was used to screen an additional 118 Australian Merino sheep biobanked at EMAI from unrelated disease investigations. This screening identified one additional carrier, suggesting a population frequency of approximately 0.6% for the *ALS2* variant in Australian Merinos. However, further genotyping is required to provide a more robust estimate of the allele frequency.

## Discussion

This study describes the identification of a likely causal variant for segmental axonopathy in Merino sheep. The 2 bp deletion encodes a predicted frameshift within a conserved region of the *ALS2* protein, resulting in a truncated protein that is 17% shorter than the mature protein. The final candidate variant was detected in heterozygous form in only two of the 935 ISGC sheep [13], not present in this database in the homozygous alternate form and is predicted to result in a frameshift and truncation of a protein with a strong functional relevance to segmental axonopathy. GWAS and homozygosity mapping supported *ALS2* as a positional candidate gene. Since genotyping of the variant has become available at EMAI, diagnostic screening of newly submitted cases has confirmed an additional affected animal from an unrelated flock, indicating continued propagation of the variant in the Australian Merino population.

A second variant in *ALS2* (XM_012142668.4:c.215A > G; p.(Asn72Ser)) was also detected but is unlikely to be pathogenic. Its high allele frequency in the ISGC dataset and presence in homozygous form in presumed healthy animals from multiple breeds, together with a SIFT score of 1, suggest that this is a tolerated protein change. Comparison with the ClinVar database shows the same position in the human reference sequence as NM_020919.4(*ALS2*):c.215G > A; p.Ser72Asn, classified as a variant of uncertain significance. In sheep, however, this represents the opposite reference allele and amino acid state, strongly suggesting that neither amino acid configuration at this position is inherently pathogenic and therefore likely representing a benign variant.

To further support the frameshifting annotation of the variant, RNA-seq data from the brain of an affected sheep was conducted and demonstrates that the 2 bp deletion impacts the reading frame of the gene. The read depth was low (4x) for a gene that is known to be highly expressed in brain tissue in other species (Human Protein Atlas proteinatlas.org) [21, 22], and while a suitable control sample could not be sourced for comparison, we speculate that the low expression in the affected animal is due to nonsense-mediated decay. We chose RNA-seq over targeted RT-PCR as it provided a more comprehensive and informative assessment of transcript structure. In addition, because the pathogenicity of the 2 bp deletion had not yet been fully established at the time of analysis, generating transcriptome-wide data ensured that alternative candidate variants or biological pathways could be explored in future studies if required.

The recently established animal variant classification guidelines [23] support that the variant is pathogenic. In these guidelines, which were adapted from the widely accepted human guidelines developed by the American College of Medical Genetics and Genomics, the frameshift variant fulfils multiple criteria to support classification of being pathogenic, including being a frameshift variant in a gene where loss of function is a known mechanism of disease in another species (PVS1), being significantly more prevalent in affected individuals compared to controls (PS4), truncating the length of the alsin protein (PM4) and being in a conserved region of the protein (PP1). The variant did not fulfil any of the nine benign classification criteria.

*ALS2* encodes the protein alsin, which regulates and activates small GTPases that control the subcellular localization of various proteins. Although alsin is widely expressed, it is particularly abundant in the nervous system, where it plays key roles in endocytosis, actin cytoskeleton maintenance, protein transport, and endosomal trafficking [21, 24]. The XP_011998058.1:p.(Leu1380Glyfs*17) variant in the ovine *ALS2* gene is predicted to disrupt the vacuolar protein sorting 9 (VPS9) domain, a guanine nucleotide exchange factor involved in activating small GTPases such as Rab5. Rab5 is crucial for early endosome fusion and maturation, serving as a key regulator in endocytic trafficking pathways [25]. Additionally, MutPred-LOF [19] analysis indicates that the frameshift variant may impact several important protein functions. Taken together, these findings, and the recessive presentation of the disease, indicate that the frameshift variant likely causes loss-of-function effects on alsin. Loss of this protein would likely impair critical cellular processes involved in neuronal survival and function, thereby contributing to the neurodegeneration in these sheep.

In human patients, variants of *ALS2* are associated with motor neuron diseases in childhood. Three syndromes are described: Infantile ascending hereditary spastic paralysis (OMIM#607,225), juvenile lateral sclerosis (OMIM#606,353) and juvenile amyotrophic lateral sclerosis (OMIM#205,100) [7]. Most inherited motor neuron diseases known as amyotrophic lateral sclerosis (ALS; OMIM Phenotypic Series PS105400) have an adult onset and a dominant mode of inheritance. The *ALS2* related early onset disorders are inherited as autosomal recessive traits. More than 100 genes are known to contain causal variants or variants associated with a susceptibility to inherited ALS, but most ALS cases are still described as sporadic [6, 24, 26]. In human patients, there are 105 pathogenic and 49 likely pathogenic allelic variants of the *ALS2* gene listed in ClinVar, of which over 20% are frameshift variants (https://www.ncbi.nlm.nih.gov/clinvar/?term=ALS2[sym]). Although motor clinical signs predominate in all forms of ALS, sensory involvement also occurs, though it is often understated in importance [27, 28]. The similarity of the disease progression in human patients with *ALS2* variants (https://www.omim.org/clinicalSynopsis/table?mimNumber=205100,606353,607225) and sheep opens the door for a new large animal model of ALS for better characterisation of the disease and evaluating new therapeutic interventions.

The Mouse Genome Database lists 26 mouse models with *ALS2* variants and alleles [29]. Among these, mice with homozygous null variants have shown a variety of clinical signs, but notably they display modest motor neuron and behavioural abnormalities and diminished coordination. Like the affected Merino sheep, there have been reports of *ALS2* knock out mice with mild motor deficits, age-dependent deficits in motor coordination and motor learning [30, 31]. Similarly, another study reported upper motor neuron clinical signs of impaired co-ordination and slowing of movement but no muscle weakness [32]. In contrast to our study and the other mouse studies mentioned, a separate study reported that *ALS2*-null mice showed no obvious motor abnormalities, although they did show a non-significant trend to decreased motor coordination [25]. *ALS2* knockdown zebrafish also demonstrated swimming defects and motor neuron degeneration [33]. While motor deficits appear common to human, mice, zebrafish and Merino sheep with *ALS2* variants, the longer life expectancy combined with severe and progressive nature of clinical disease in Merino sheep may be more suitable as a model of human disease compared to mice.

Although there are some similarities in clinical presentation and disease progression between mice, sheep, and humans, there are notable variations in the morphologic changes observed in mouse models with *ALS2* variants compared to Merino sheep. *ALS2* mouse models have presented with a range of findings, including a subtle decrease in the size of cortical motor neurons [25], axonal degeneration in the lateral spinal cord [30], and age-associated decreases in Purkinje cells and changes secondary to subclinical deficits in spinal motor neurons [31]. One study reports degenerative changes in distal corticospinal tracts on special stains but no pathologic changes in motor neurons [29]. In other models, there are no obvious neuropathologic abnormalities, but neurons show increased susceptibility to oxidative stress [28]. The variation in morphologic changes observed in mice may be partly due to strain differences or the extent of gene inactivation [29–31].

Differences in clinical phenotypes and severity between mice and humans with *ALS2* variants are thought to arise from several factors. These include the longer length of motor neurons in humans, the lack of direct synaptic connections between upper and lower motor neurons in mice, and anatomical differences in the distribution of ascending and descending spinal tracts between the two species. Other contributing factors could include differences in the roles of the upper motor system, compensatory mechanisms, and potential gene redundancy [25, 28–30].

Research towards the identification of a causal variant has been ongoing since the advent of advanced genetic technologies in sheep. However, these efforts were using smaller sample numbers for both GWAS and WGS. The collaboration between the Australian and New Zealand research teams meant the number of affected animals could increase when DNA technology cost decreased. Two unpublished GWAS studies conducted by our research teams (one in Australia, one in New Zealand) have been performed on much smaller cohorts and based on 50 K SNP chip data. There were only 51 SNP markers in the *ALS2* gene region (± 1 Mb), compared to 552 SNP markers on the high density 600 K chip, meaning the GWAS peak was more easily distinguishable when increased sample numbers provided stronger association p-values. Additionally, gene annotation of the ovine genome has improved since the commencement of initial investigations.

On histologic blinded review of cases, the previously reported large, foamy pale eosinophilic axonal swellings were found to be the defining histologic feature of segmental axonopathy [2–4]. While white matter vacuolation and non-specific Wallerian degeneration can also occur in segmental axonopathy, the presence of large foamy axonal swellings was the key feature that allowed differentiation from an array of causes of Wallerian degeneration.

Histologic lesions and axonal degenerative changes in the brain and spinal cord are key to diagnosing segmental axonopathy. Large foamy axonal swellings were present in both the spinal cord and brain in all sheep with available formalin-fixed, paraffin-embedded tissue. While axonal swellings are typically widespread in white matter, some reports have noted empty white matter vacuoles as a predominant but non-specific histologic change [2]. However, the concordance between the *ALS2* genotypes and the histological changes indicates that the characteristic granular eosinophilic swellings are critical for an accurate diagnosis. Therefore, it is essential to sample the appropriate areas of the brain and spinal cord, with the trigeminal ganglion and sensory root being the most effective locations for identifying these swellings.

In segmental axonopathy of Merino sheep, the dominant histological lesions involved the dorsal ganglia roots, dorsal funiculus and gracile fasciculus of spinal cord, the pons and the ascending cerebellar tracts, although motor tracts were also involved [4, 5]. The segmental nature of the disease is associated with multiple vesicles derived from localised in-pouching and degeneration of myelin. This is likely due to defects in the underlying cytoskeleton and axolemma as previously postulated [4], reflecting the importance of alsin in maintaining the actin cytoskeleton.

To our knowledge, this disease is restricted to fine-wool Merino sheep, of which there are an estimated 31 million breeding ewes in Australia [34] and over 3 million Merinos in New Zealand [35]. Fine wool Merinos are a type of Merino sheep that produces wool of 19.5 microns or less that is used to produce high quality soft fabrics and clothes. While our findings support that the variant is present in multiple flocks in Australia and New Zealand, genotyping of more fine wool Merinos as well as other sheep is needed to accurately estimate the allele frequency and predict how many sheep are heterozygous. The significance of finding multiple unrelated Merino flocks across two countries with homozygous affected animals, as well as heterozygotes from other flocks not yet reported to have disease, suggests that the availability of a DNA test will be of substantial benefit to Merino breeders globally. As this slowly progressive neurological disease is often not recognised as an inherited condition and often leads to accidental death in extensive management systems, implementation of DNA testing will reduce the risk of affected animals being born and thus improve animal welfare. DNA testing focusing on identification of clinically unaffected heterozygous animals, especially rams, is critical in flocks where the disorder is present. Due to the relatively high frequency of this variant in the Merino population, inclusion of the variant in genotyping panels used by industry for genomic selection should be considered to identify at-risk flocks.

Given the genetic diagnosis of segmental axonopathy and its potential as a model for similar human disorders, further detailed clinical, molecular, and histological investigations are warranted. Comparative studies of Wallerian degeneration and distal degenerative axonopathy in peripheral nerves could provide valuable insights, and advanced diagnostic techniques such as immunohistochemistry, RT-qPCR, and Western blotting would enable more precise characterisation of the *ALS2* variant phenotype in sheep, including transcript quantification and assessment of protein expression. This study was limited by the lack of age-matched control tissue, as well as the absence of suitable fresh-frozen samples or cell lines from affected or control animals, precluding such analyses. Addressing these limitations in future work will be essential to confirm the functional impact of the frameshift variant and establish the suitability of these sheep as a disease model. Nevertheless, identification of the likely causal variant has already facilitated the development of a DNA-based diagnostic test, providing an immediate and practical tool to help mitigate the economic, production, and welfare impacts of this disorder on the Merino industry.

## Conclusions

This study identifies a likely causal frameshift variant in the *ALS2* gene, associated with segmental axonopathy in Merino sheep. The variant introduces a premature stop codon, resulting in truncation of the alsin protein and disruption of a highly conserved functional domain likely essential for neuronal health. Strong evidence from genome sequencing, genotype–phenotype correlation, GWAS and homozygosity mapping, and RNA-seq supports its pathogenicity. Histological examination confirms a consistent neurodegenerative profile marked by distinctive axonal swellings, establishing robust diagnostic criteria for the disease.

The findings are of significant importance to the Merino industry, as the variant is present in multiple unrelated flocks across Australia and New Zealand. The development of a DNA test for this variant offers a practical and immediate tool for breeders to screen carriers and reduce the risk of disease propagation, ultimately improving animal welfare and productivity.

Beyond its agricultural impact, this work has broader relevance as it introduces Merino sheep as a promising large-animal model for human juvenile motor neuron diseases caused by *ALS2* mutations. Compared to existing rodent models, sheep offer advantages in disease progression, size, and lifespan, which may facilitate more translatable insights into the pathogenesis and treatment of early-onset amyotrophic lateral sclerosis. The study also underscores the value of integrating genomic, transcriptomic, and histopathological data to characterise rare diseases in livestock and their potential analogues in human health.

## Supplementary Information

Additional file 1. Table S1. Description: Genomic positions and characteristics of 79 variants identified through whole genome sequencing following zygosity filtering and predicted protein effect analysis. Likely causal variant identified from this study is highlighted in red.

Additional file 2. Description: List of the top 10 SNPs most significantly associated with segmental axonopathy in sheep, all located on chromosome 2 and flanking the *ALS2* gene region (chr2:202,925,505 - 202,987,007 on Oar v3.1)

Additional file 3. Description: RNA-seq data from the cerebellar peduncle of a single affected animal, confirming the *ALS2* transcript annotation and the predicted effect of the 2 bp deletion (XM_012142668.4:c.4138_4139del), resulting in a frameshift and premature stop codon (XP_011998058.1:p.(Leu1380Glyfs*17)). Panels A and B display two zoom levels (5.3 kbp and 110 bp), with the variant indicated by a red arrow in Panel A and positioned near a splice acceptor site in Panel B.

## Data Availability

All whole genome sequence data was deposited as Bioproject PRJNA1015710 (ID 1015710—BioProject—NCBI).

## References

[1] Nicholas F., Tammen I., Sydney Informatics Hub. Online Mendelian Inheritance in Animals (OMIA), The University of Sydney, 2025.

[2] Harper PA, Duncan DW, Plant JW, Smeal MG. Cerebellar abiotrophy and segmental axonopathy: two syndromes of progressive ataxia of Merino sheep. Aust Vet J. 1986;63:18–21. 3954688 10.1111/j.1751-0813.1986.tb02865.x

[3] Hartley WJ, Loomis LN. Murrurundi disease: an encephalopathy of sheep. Aust Vet J. 1981;57:399–400. 7342953 10.1111/j.1751-0813.1981.tb00541.x

[4] Jolly RD, Johnstone AC, Williams SD, Zhang K, Jordan TW. Segmental axonopathy of Merino sheep in New Zealand. N Z Vet J. 2006;54:210–7. 17028657 10.1080/00480169.2006.36699

[5] Windsor PA. Ultrastructural findings in ovine segmental axonopathy of Merino sheep. Aust Vet J. 2006;84:169–72. 16739526 10.1111/j.1751-0813.2006.tb12772.x

[6] Kenna KP, McLaughlin RL, Byrne S, Elamin M, Heverin M, Kenny EM, et al. Delineating the genetic heterogeneity of ALS using targeted high-throughput sequencing. J Med Genet. 2013;50:776–83. 23881933 10.1136/jmedgenet-2013-101795 PMC3812897

[7] Miceli M, Exertier C, Cavaglia M, Gugole E, Boccardo M, Casaluci RR, et al. ALS2-related motor neuron diseases: from symptoms to molecules. Biology. 2022;11:77. 35053075 10.3390/biology11010077 PMC8773251

[8] Bolger AM, Lohse M, Usadel B. Trimmomatic: a flexible trimmer for Illumina sequence data. Bioinformatics. 2014;30:2114–20. 24695404 10.1093/bioinformatics/btu170 PMC4103590

[9] Vasimuddin M., Misra S., Li H., Aluru S., Efficient Architecture-Aware Acceleration of BWA-MEM for Multicore Systems, in 2019 IEEE International Parallel and Distributed Processing Symposium (IPDPS), 2019; 314–324.

[10] Poplin R, Ruano-Rubio V, DePristo MA, Fennell TJ, Carneiro MO, Van der Auwera GA, et al. Scaling accurate genetic variant discovery to tens of thousands of samples. bioRxiv. 2018. 10.1101/201178.

[11] Caetano-Anolles D., Hard-filtering germline short variants., in: GATK Technical Documentation / Algorithms [on line] (2023) https://gatk.broadinstitute.org/hc/en-us/articles/360035890471-Hard-filtering-germline-short-variants. Accessed 15 Oct 2024.

[12] Cingolani P, Platts A, Wang le L, Coon M, Nguyen T, Wang L, et al. A program for annotating and predicting the effects of single nucleotide polymorphisms, SnpEff: SNPs in the genome of *Drosophila melanogaster* strain w1118; iso-2; iso-3. Fly (Austin). 2012;6(2):80–92. 22728672 10.4161/fly.19695 PMC3679285

[13] Daetwyler H., Kijas J., McWilliam S., Clarke S., Brauning R., Sheep genome variants V2, Commonwealth Scientific and Industrial Research Organisation (CSIRO), 2019.

[14] Chang CC, Chow CC, Tellier LC, Vattikuti S, Purcell SM, Lee JJ. Second-generation PLINK: rising to the challenge of larger and richer datasets. Gigascience. 2015;4(2015):7. 25722852 10.1186/s13742-015-0047-8 PMC4342193

[15] Turner SD. qqman: an R package for visualizing GWAS results using Q-Q and Manhattan plots. bioRxiv. 2014. 10.1101/005165.

[16] Dobin A, Davis CA, Schlesinger F, Drenkow J, Zaleski C, Jha S, et al. STAR: ultrafast universal RNA-seq aligner. Bioinformatics. 2013;29:15–21. 23104886 10.1093/bioinformatics/bts635 PMC3530905

[17] Robinson JT, Thorvaldsdottir H, Winckler W, Guttman M, Lander ES, Getz G, et al. Integrative genomics viewer. Nat Biotechnol. 2011;29:24–6. 21221095 10.1038/nbt.1754 PMC3346182

[18] Edgar RC. MUSCLE: multiple sequence alignment with high accuracy and high throughput. Nucleic Acids Res. 2004;32:1792–7. 15034147 10.1093/nar/gkh340 PMC390337

[19] Pagel KA, Pejaver V, Lin GN, Nam HJ, Mort M, Cooper DN, et al. When loss-of-function is loss of function: assessing mutational signatures and impact of loss-of-function genetic variants. Bioinformatics. 2017;33:i389–98. 28882004 10.1093/bioinformatics/btx272 PMC5870554

[20] Vaser R, Adusumalli S, Leng SN, Sikic M, Ng PC. Sift missense predictions for genomes. Nat Protoc. 2016;11:1–9. 26633127 10.1038/nprot.2015.123

[21] Hadano S, Hand CK, Osuga H, Yanagisawa Y, Otomo A, Devon RS, et al. A gene encoding a putative GTPase regulator is mutated in familial amyotrophic lateral sclerosis 2. Nat Genet. 2001;29:166–73. 11586298 10.1038/ng1001-166

[22] Sjostedt E, Zhong W, Fagerberg L, Karlsson M, Mitsios N, Adori C, et al. An atlas of the protein-coding genes in the human, pig, and mouse brain. Science. 2020. 10.1126/science.aay5947. 32139519 10.1126/science.aay5947

[23] Boeykens F, Abitbol M, Anderson H, Casselman I, de Citres CD, Hayward JJ, et al. Development and validation of animal variant classification guidelines to objectively evaluate genetic variant pathogenicity in domestic animals. Front Vet Sci. 2024;11:1350474. 10.3389/fvets.2024.1497817 PMC11656590 39703406

[24] Brettschneider J, Del Tredici K, Toledo JB, Robinson JL, Irwin DJ, Grossman M, et al. Stages of pTDP-43 pathology in amyotrophic lateral sclerosis. Ann Neurol. 2013;74:20–38. 23686809 10.1002/ana.23937 PMC3785076

[25] Hadano S, Benn SC, Kakuta S, Otomo A, Sudo K, Kunita R, et al. Mice deficient in the Rab5 guanine nucleotide exchange factor ALS2/alsin exhibit age-dependent neurological deficits and altered endosome trafficking. Hum Mol Genet. 2006;15:233–50. 16321985 10.1093/hmg/ddi440

[26] Hardiman O, Al-Chalabi A, Chio A, Corr EM, Logroscino G, Robberecht W, et al. Amyotrophic lateral sclerosis. Nat Rev Dis Primers. 2017;3:17071. 28980624 10.1038/nrdp.2017.71

[27] Riancho J, Paz-Fajardo L. Lopez de Munain A., Clinical and preclinical evidence of somatosensory involvement in amyotrophic lateral sclerosis. Br J Pharmacol. 2021;178:1257–68. 32673410 10.1111/bph.15202

[28] Rubio MA, Herrando-Grabulosa M, Navarro X. Sensory involvement in amyotrophic lateral sclerosis. Int J Mol Sci. 2022. 10.3390/ijms232415521. 36555161 10.3390/ijms232415521 PMC9779879

[29] Blake JA, Baldarelli R, Kadin JA, Richardson JE, Smith CL, Bult CJ. Mouse Genome Database G Mouse Genome Database (MGD): Knowledgebase for mouse-human comparative biology. Nucleic Acids Res. 2021;49:D981–7. 33231642 10.1093/nar/gkaa1083 PMC7779030

[30] Cai H, Lin X, Xie C, Laird FM, Lai C, Wen H, et al. Loss of ALS2 function is insufficient to trigger motor neuron degeneration in knock-out mice but predisposes neurons to oxidative stress. J Neurosci. 2005;25:7567–74. 16107644 10.1523/JNEUROSCI.1645-05.2005 PMC2364727

[31] Deng HX, Zhai H, Fu R, Shi Y, Gorrie GH, Yang Y, et al. Distal axonopathy in an alsin-deficient mouse model. Hum Mol Genet. 2007;16:2911–20. 17855450 10.1093/hmg/ddm251

[32] Yamanaka K, Miller TM, McAlonis-Downes M, Chun SJ, Cleveland DW. Progressive spinal axonal degeneration and slowness in ALS2-deficient mice. Ann Neurol. 2006;60:95–104. 16802286 10.1002/ana.20888

[33] Gros-Louis F, Kriz J, Kabashi E, McDearmid J, Millecamps S, Urushitani M, et al. Als2 mRNA splicing variants detected in KO mice rescue severe motor dysfunction phenotype in Als2 knock-down zebrafish. Hum Mol Genet. 2008;17:2691–702. 18558633 10.1093/hmg/ddn171

[34] Meat & Livestock Australia. National flock numbers continue to grow. 2022 Apr 21. https://www.mla.com.au/news-and-events/industry-news/national-flock-numbers-continue-to-grow/. Accessed 15 Oct 2024.

[35] Rare Breeds Conservation Society of New Zealand Incorporated. How Many Sheep? Sheep Numbers in New Zealand, 2005. https://www.rarebreeds.co.nz/sheepnumbers.html Accessed 15 Oct 2024.

