# Supplementary material for: A novel frameshift variant in ALS2 associated with segmental axonopathy in Merino sheep
Source: Genet Sel Evol. 2025 Oct 23;57:60. doi: 10.1186/s12711-025-01005-w (PMC12551161; doi:10.1186/s12711-025-01005-w)

Figure S1: **RNA-sequence alignment data representing the *ALS2* transcript, visualised in the Integrative Genomics Viewer (IGV):** RNA-seq data from the cerebellar peduncle of a single affected animal, confirming the *ALS2* transcript annotation and the predicted effect of the 2 bp deletion (XM_012142668.4:c.4138_4139del), resulting in a frameshift and premature stop codon (XP_011998058.1:p.(Leu1380Glyfs*17)). Panels A and B display two zoom levels (5.3 kbp and 110 bp), with the variant indicated by a red arrow in Panel A and positioned near a splice acceptor site in Panel B.

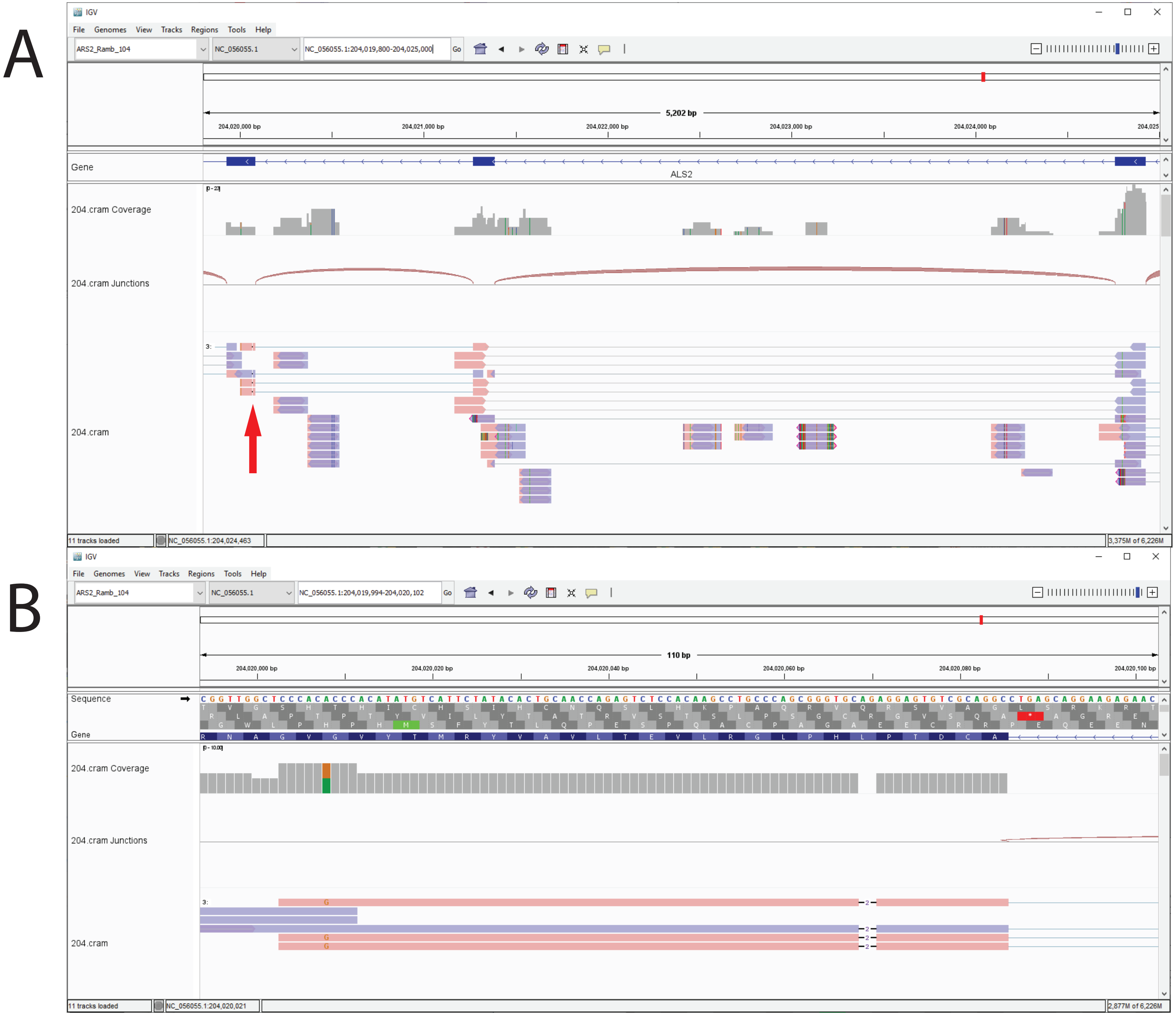

Supplement: Supplementary file 3 — Additional file 3. Description: RNA-seq data from the cerebellar peduncle of a single affected animal, confirming the ALS2 transcript annotation and the predicted effect of the 2 bp deletion (XM_012142668.4:c.4138_4139del), resulting in a frameshift and premature stop codon (XP_011998058.1:p.(Leu1380Glyfs*17)). Panels A and B display two zoom levels (5.3 kbp and 110 bp), with the variant indicated by a red arrow in Panel A and positioned near a splice acceptor site in Panel B. [file 12711_2025_1005_MOESM3_ESM.pdf]
